# Juglone Attenuates CCl₄‐Induced Hepatic Injury in Mice With Associated Modulation of Oxidative Stress and Inflammasome‐Related mRNA Expression

**DOI:** 10.1155/bmri/8905392

**Published:** 2026-07-21

**Authors:** Aamir Sohail, Raza Sufyan, Muhammad Asim, Mehroz Khan, Aleena Altaf, Muhammad Ehsan Ul Haq, Maimoona Arshad, Atif Ali Khan Khalil, Imran Ullah

**Affiliations:** ^1^ Department of Biochemistry, Faculty of Biological Sciences, Quaid-i-Azam University, Islamabad, Pakistan, qau.edu.pk; ^2^ Department of Biotechnology, Yeungnam University, Gyeongsan, Republic of Korea, yu.ac.kr; ^3^ Lab of Reproductive Biotechnology, Gyeongsang National University, Jinju, Republic of Korea, gnu.ac.kr

**Keywords:** carbon tetrachloride (CCl₄), fibrosis, juglone, liver injury, NLRP3 inflammasome, pyroptosis

## Abstract

Carbon tetrachloride–induced liver injury is a well‐established model of toxic hepatitis, characterized by marked oxidative and inflammatory damage. It remains a relevant experimental system because broadly effective therapeutic options are still limited. Juglone, a natural naphthoquinone derived from *Reynoutria japonica*, has demonstrated antioxidant and anti‐inflammatory potential. However, mouse evidence combining standard liver injury measures with stress‐ and cell‐death gene profiling remains limited. Building on juglone studies in injury and fibrosis, we tested juglone in a CCl_4_ model. Male C57BL/6 mice were assigned to normal, vehicle, CCl₄, silymarin (200 mg/kg), and juglone (8 mg/kg) groups, and CCl₄ (in corn oil) was administered intraperitoneally. Endpoints included body weight, serum liver enzymes and lipid profile, H&E histology, and RT‐qPCR panels for inflammatory, pyroptosis‐associated, ER stress and autophagy‐linked, apoptotic, oxidative‐stress, and profibrotic transcripts. CCl₄ exposure was associated with weight loss, enzyme elevation, dyslipidemia, and architectural disruption, whereas juglone treatment was associated with improved biochemical indices and histology alongside coordinated downregulation of inflammatory/pyroptosis‐related transcripts (including *Il-1β*, *Il-6*, *Nlrp3*, and *Gsdmd*) and moderation of ER stress/autophagy and apoptosis‐linked transcripts, with downregulation of profibrotic transcripts (*Acta2*, *Mmp2*, and *Tgfβ1*). In this mouse CCl₄ model, juglone treatment was associated with reduced serum and tissue injury signatures together with changes in stress and inflammation‐linked transcriptional programs. Because the molecular findings are primarily based on RT‐qPCR, these results are best interpreted as gene‐level associations compatible with modulation of oxidative stress, inflammasome‐ and pyroptosis‐related mRNA expression, ER stress responses, apoptosis‐linked transcripts, and remodeling‐associated transcripts, rather than as confirmed pathway inhibition or established antifibrotic activity. These data support juglone as a hepatoprotective candidate and identify protein‐level and functional validation as important next steps.

## 1. Introduction

The liver is a major organ which plays an essential role in many metabolic and detoxification processes. It measures approximately 2% of body weight, and its size varies with physiological parameters such as sex [1]. Hepatic blood supply via the portal vein and hepatic artery supports carbohydrate, protein and lipid metabolism, bile production, and detoxification of xenobiotics [2–3]. In this setting, parenchymal hepatocytes act together with Kupffer cells and hepatic stellate cells (HSCs) to coordinate metabolic and immune functions [2, 3]. A highly relevant task of the hepatic system is the biotransformation of lipophilic chemicals such as carbon tetrachloride (CCl_4_). In Phase I metabolism, CCl_4_ is converted into reactive radicals through cytochrome P450 enzymes, primarily CYP2E1, which promotes the generation of reactive metabolites and reactive oxygen species (ROS) within hepatocytes [4, 5]. Liver disease has a large global burden, and persistent injury can culminate in fibrosis, cirrhosis, and hepatocellular carcinoma [6]. However, available therapies rarely stop injury or reverse fibrosis, which highlights the need for better hepatoprotective agents [7]. Accordingly, the CCl_4_ model remains widely used to study chemically induced liver injury and to evaluate candidate hepatoprotective interventions [8].

Furthermore, CYP2E1‐derived radicals initiate lipid peroxidation and membrane damage, leading to hepatocyte necrosis and apoptosis, whereas Kupffer cell and HSC activation amplifies inflammatory signaling and profibrogenic remodeling [9, 10]. Oxidative stress which is defined as an imbalance between ROS and antioxidant defenses is central in this process [11], and ROS‐driven macromolecular damage can intensify inflammation and cell death [12]. The Nrf2–Keap1 (nuclear factor erythroid 2–related factor 2 and Kelch‐like ECH‐associated protein 1) pathway coordinates Phase II antioxidant defenses (e.g., NQO1 and HO‐1), and disruption of this control worsens injury and favors fibrotic progression [12]. In parallel, ROS, mitochondrial dysfunction, and endoplasmic reticulum (ER) stress can activate the NLRP3 (NOD‐like receptor family pyrin domain containing 3) inflammasome, promoting maturation of IL‐1*β* and IL‐18 and facilitating HSC activation [13]. ER stress pathways (PERK–eIF2*α*, IRE1*α*, and ATF6) also intersect with inflammasome signaling and pyroptosis [14, 15], and chronic injury can shift wound‐healing toward excessive extracellular matrix deposition, with activated HSCs expressing *α*‐smooth muscle actin (*α*‐SMA) and producing collagen under TGF‐*β* and Smad signaling [10, 16].

Despite the established importance of oxidative stress in CCl_4_ toxicity, a practical gap remains, and many hepatoprotective strategies primarily target oxidative injury yet fail to address how oxidative stress couples with ER stress and inflammasome‐linked inflammatory cell death programs that can sustain cytokine release and reinforce profibrogenic signaling [7, 12–15]. This incomplete targeting may partly explain why improvement in classical liver enzymes does not always translate into durable suppression of tissue remodeling in chronic toxic injury [7, 10, 16].

Juglone (5‐hydroxy‐1,4‐naphthoquinone), a redox‐active naphthoquinone reported from *Reynoutria japonica*, has been associated with antioxidant, anti‐inflammatory, and anti‐fibrotic effects, including improved antioxidant defenses and reduced fibrotic marker expression [17–18]. Based on its quinone structure and redox activity, we hypothesized that juglone may attenuate CCl_4_‐induced liver injury not only by limiting oxidative stress but also by modulating ER‐stress signaling and suppressing NLRP3‐linked inflammatory programs, thereby reducing downstream pathways that promote remodeling.

Previous work examined juglone‐related hepatoprotection in rat fibrosis paradigms, focusing on antioxidant status and fibrosis markers such as *α*‐SMA and collagen III, or in CCl_4_‐treated rats using walnut leaf extracts reporting changes in liver enzymes, SOD, catalase, and histology [18, 19]. However, in our study, we evaluated purified juglone in a mouse CCl_4_‐induced liver injury model, focusing on a proposed link between redox imbalance, ER stress, and NLRP3‐driven pyroptosis. We assessed serum biochemistry, histopathology, and an RT‐qPCR gene panel covering inflammatory, oxidative, ER‐stress, autophagy, apoptotic, and fibrotic pathways. Juglone mitigated CCl_4_‐related liver injury by reducing oxidative stress while also dampening ER‐stress signaling and NLRP3‐associated inflammatory cell death. These effects were reflected in improved body‐weight recovery and gross liver appearance, lower biochemical injury markers and ROS signals, better preservation of lobular architecture, and gene‐expression shifts toward the control profile across inflammation, pyroptosis, stress response, apoptosis, and tissue remodeling.

## 2. Materials and Methods

### 2.1. Animals and Experimental Conditions

Male C57BL/6 mice (age 6–8 weeks, body weight 22–28 g) were obtained from the Primate Facility, National Institute of Health (NIH), Islamabad. Animals were acclimatized for 1 week in standard cages at the Department of Biochemistry, Quaid‐i‐Azam University, Islamabad, under Specific Pathogen‐Free (SPF) conditions (22°C ± 2°C; 50*%*–60*%* humidity; 12‐h light/12‐h dark cycle). During acclimatization and experimentation, mice had ad libitum access to standard rodent chow and tap water.

### 2.2. Ethics Statement

The animal experiments received approval from the Animal Bioethics Committee of Quaid‐i‐Azam University (BEC‐FBS‐QAU2020‐271), Islamabad, Pakistan. The procedures adhered to the relevant guidelines and regulations, including institutional policies and the US National Institutes of Health Guide for the Care and Use of Laboratory Animals. The study was conducted and reported in accordance with the ARRIVE 2.0 (2020) guidelines for in vivo research.

### 2.3. Experimental Design and Timeline

The liver injury was induced by repeated administration of CCl₄ over a 3‐week period. After this, CCl_4_ dosing was halted, and the following 2‐week period was used to assess the effects of the intervention during the early postinjury phase. This methodology aligns with the well‐established CCl_4_ mouse model, in which repeated dosing leads to a prolonged wave of hepatic oxidative damage and inflammatory remodeling, which remains detectable even after the final toxin exposure and can be captured at specific harvest time points [20].

### 2.4. Grouping and Treatment Regimen

The study included five groups with six animals in each group: normal, CCl₄, vehicle, silymarin, and juglone. The experimental design was a postinjury recovery model. In this design, liver injury was first induced through repeated CCl₄ administration during the first 3 weeks of the experiment. After completion of the injury‐induction phase, CCl₄ administration was stopped, and the following 2 weeks were used to assess the postinjury recovery effects of silymarin and juglone. The total experimental duration was 5 weeks, corresponding to 35 days.

During the induction phase, mice in the CCl₄ group received CCl_4_ prepared as a 30% (v/v) solution in corn oil by intraperitoneal injection at a dose of 0.5 mL/kg body weight, administered twice per week for 3 weeks to reliably induce hepatic injury. This treatment was administered twice per week for 3 weeks (on alternate days, specifically Days 1 and 4 of each week) to establish the liver injury model. The vehicle group received corn oil by i.p. injection at the same final injection volume and on the same induction schedule as the CCl₄ group to match the toxin solvent exposure. The normal group did not receive CCl₄ and received saline by i.p. injection during the postinjury phase to match handling and injection exposure.

After the 3‐week CCl₄ induction period, no further CCl₄ was administered. The silymarin group received the same CCl₄ induction protocol and was then treated with silymarin at 200 mg/kg by i.p. injection twice per week for 2 weeks (on alternate days, specifically Days 1 and 4 of each week), with four injections in total. The juglone group received the same CCl₄ induction protocol and was then treated with juglone at 8 mg/kg, prepared in 1% dimethyl sulfoxide (DMSO), by i.p. injection twice per week for 2 weeks (on alternate days, specifically Days 1 and 4 of each week), with four injections in total. The vehicle group received corn oil during the CCl₄ induction phase, with 1% DMSO added during the postinjury period to match the juglone formulation, whereas the normal group received four saline injections during the same postinjury period to match handling and injection exposure. The postinjury injections were administered on Days 22, 25, 29, and 32, and the experiment was completed on Day 35. Body weight was recorded daily throughout the 35‐day study period.

### 2.5. Fasting and Terminal Sampling

Animals were fasted prior to terminal blood collection to reduce variability in lipid readouts and were euthanized at the end of the 35‐day experimental period, and blood and liver tissues were collected for biochemical, histological, and molecular analyses.

### 2.6. Terminal Anesthesia, Blood/Serum Collection, and Euthanasia

At the end of the study, mice were deeply anesthetized with ketamine (100 mg/kg, i.p.) and xylazine (10 mg/kg, i.p.), and depth of anesthesia was confirmed by the absence of the pedal reflex. Approximately 1 mL of blood was collected by cardiac puncture into clot‐activator vacutainers (Xinle) and allowed to clot at room temperature for 30 min. Samples were centrifuged at 6000 rpm for 10 min at 4°C (Hermile Labortechnit GmbH, Siemensstr‐25, D‐78564, Wehingen), and serum was aliquoted into RNase/DNase‐free tubes and stored at −20°C. Animals were euthanized immediately after sample collection by anesthetic overdose (ketamine/xylazine; cumulative ≥ 300/30 mg/kg, i.p.), followed by cervical dislocation as a confirmatory physical method, in accordance with current veterinary guidelines.

### 2.7. Dissection and Organ Harvesting

Following blood collection, dissection was performed under sterile and chilled conditions using a midline abdominal incision to expose visceral organs. The liver was excised, rinsed with phosphate‐buffered saline (PBS), washed with chilled double‐distilled water, and examined macroscopically for signs of hepatotoxicity such as discoloration, fibrosis‐like surface changes, or lesions. Tissue samples were placed in 10% neutral buffered formalin for fixation, whereas others were snap‐frozen in liquid nitrogen for molecular analysis. The frozen tissue was then stored at −80°C.

### 2.8. Biochemical Assessments

Serum alanine aminotransferase (ALT), aspartate aminotransferase (AST), and alkaline phosphatase (ALP) were quantified using commercially available diagnostic kits (AMP Diagnostics) following the manufacturer′s protocols. Lipid profile parameters, including triglycerides (TAG), total cholesterol (TC), high‐density lipoprotein cholesterol (HDL‐C), very‐low‐density lipoprotein (VLDL), and low‐density lipoprotein cholesterol (LDL‐C), were measured enzymatically (AMP Diagnostics). Serum reactive oxygen metabolites were measured using the derivatives of reactive oxygen metabolites (d‐ROMs) spectrophotometric assay, following the principle and methods described by Hayashi et al. [21]. Serum samples were mixed with chromogenic reagent and incubated at 37°C, and kinetic absorbance at 505 nm was recorded on a microplate reader at 15, 30, 45, 60, 120, and 240 s in a similar fashion to protocols reported in recent oxidative stress studies [22].

### 2.9. Histopathological Analysis via H&E Staining

Formalin‐fixed liver tissues were processed through a graded ethanol series, cleared, and embedded in paraffin. Sections (4 *μ*m) were cut on a rotary microtome (KD202, China), mounted on glass slides, and stained with hematoxylin and eosin (H&E) to assess hepatocellular necrosis, inflammatory infiltration, and overall tissue architecture. The slides were examined using a bright field microscope (CX41, Olympus, Japan), with representative fields captured for documentation. For quantitative assessment, one stained liver section from each animal was analyzed, and five nonoverlapping lobular fields were selected per section, avoiding large vessels, folded areas, and damaged edges. Images were coded before analysis, and scoring was performed without group identification. Hepatocyte number was quantified using ImageJ and normalized to the mean value of the normal group. Lobular inflammation was assessed semiquantitatively based on inflammatory‐cell infiltration and lobular architectural disruption, and the final score for each animal was calculated from the selected fields. Group‐level statistics were performed using GraphPad Prism.

### 2.10. RNA Extraction

Total RNA was extracted from 50–100 mg of snap‐frozen liver tissue using an Invitrogen RNA isolation kit (Thermo Fisher Scientific, United States) according to the manufacturer′s protocol. For the liver tissue, it was first powdered in liquid nitrogen and homogenized in a lysis buffer containing *β*‐mercaptoethanol. The tissue was then processed through chloroform phase separation and column‐based purification, following the kit instructions. RNA concentration and purity were assessed using a NanoDrop spectrophotometer (Colibri; Berthold Detection Systems GmbH, Germany), and samples with A260/280 between 1.8 and 2.0 were taken forward for cDNA synthesis.

### 2.11. cDNA Synthesis

One microgram of purified RNA from each animal was reverse‐transcribed into complementary DNA (cDNA) using the Thermo Scientific RevertAid First Strand cDNA Synthesis Kit (Thermo Fisher Scientific, United States) in a 20‐*μ*L reaction volume, following the manufacturer′s protocol. The reaction mixture contained reverse transcriptase, reaction buffer, dNTPs, RNase inhibitor, and oligo (dT) primers, and reverse transcription was performed at 42°C for 60 min followed by enzyme inactivation at 70°C for 5 min. Synthesized cDNA was stored at −20°C until downstream RT‐qPCR analysis.

### 2.12. Quantitative Real‐Time PCR (RT‐qPCR)

RT‐qPCR was performed using the MyGo Pro real‐time PCR system (IT‐IS Life Science Ltd.) with SYBR Green chemistry. Each 10‐*μ*L reaction contained SYBR Green Master Mix, gene‐specific forward and reverse primers (10 *μ*M), and diluted cDNA (1:10), and cycling was carried out with an initial denaturation step followed by 40 amplification cycles using the annealing/extension conditions described in the original protocol. Primer sequences are provided in Table 1. *β-actin* was used as the internal control for normalization. Relative mRNA expression was calculated using the 2^−*ΔΔ*Ct^ method, and all comparisons were generated from biological replicates at the animal level with each sample measured in technical triplicate wells per gene; technical triplicates were averaged to produce one value per animal per target before group‐level statistics.

**Table 1:** List of primers for RT‐qPCR.

Gene	5 ^′^to 3 ^′^sequence	TM (°C)	Amplicon size	Species
*Txnip*	**F:** TTCCTGTCCAGTGTTGGGA	60.1	115 bp	Mouse
	**R:** CTGCACAGTTCTCAGGTGGA	60.0		
*Acta2*	**F:** GCCTCCAGTTCCTTTCCAA	60.2	121 bp	Mouse
	**R:** ATCAGTGTTGCTAGGCCAGG	60.3		
*Atf-6α*	**F:** TTAGAGTGCCCGAAGCCA	60.5	99 bp	Mouse
	**R:** CCGATCTTCCCACTTCCAC	60.5		
*Mmp-2*	**F:** TGGTGCTCCACTCTTCTGG	60.0	90 bp	Mouse
	**R:** GCCCTCCTAAGCCAGTCTCT	60.0		
*Gsdmd*	**F:** CCCTTCCCACAACATCTCC	60.3	77 bp	Mouse
	**R:** CTTGGCTTCCCAAAGGCT	60.3		
*Perk*	**F:** ACTTCAAGGAAAGGGCTGTGT	60.2	62 bp	Mouse
	**R:** AGTCTTGGGACACCGACAAG	60.2		
*Nf-kβ 1*	**F:** GCGTCCTTTCTTGGTTCTGA	60.4	114 bp	Mouse
	**R:** GCTCAAGACACTGCACCTGA	60.2		
*Jnk*	**F:** GGGTGCTGATGCTTTCAGAT	60.2	98 bp	Mouse
	**R:** CAGAGGGTACACGGCTTCC	60.7		
*Il-1β*	**F:** AGGGGACATTAGGCAGCAC	60.1	78 bp	Mouse
	**R:** AGTGCGGGCTATGACCAA	60.2		
*Ern1 (Ire1)*	**F:** GCAGCCTTATCCACACTGCT	60.4	64 bp	Mouse
	**R:** AACACACAGGGGAACAGGAG	60.0		
*Il-18*	**F:** GGGAGGGTTTGTGTTCCAG	62.3	90 bp	Mouse
	**R:** GCAGCCTCGGGTATTCTGT	62.3		
*Il-6*	**F:** CGGCAAACCTAGTGCGTTAT	60.2	63 bp	Mouse
	**R:** TCTGACCACAGTGAGGAATGTC	60.2		
*Il-10*	**F:** CCAGGGATCTTAGCTAACGGA	62.6	61 bp	Mouse
	**R:** TTCGGAGAGAGGTACAACGAG	62.7		
*Cyp2e1*	**F:** TATCGACCTCAGCCCTGTTAC	59.2	102 bp	Mouse
	**R:** GGATAATGATGGGCAGCAG	59.0		
*Cox-2*	**F:** GACTTGCCAGGCTGAACTTC	60.0	96 bp	Mouse
	**R:** GCTCACGAGGCCACTGATA	60.0		
*Bcl-2*	**F:** GGCTCCCTTCATGAAATCCT	60.4	101 bp	Mouse
	**R:** AGAACCCCTGTCTCCAAAGG	60.5		
*Tgf-β1*	**F:** GGAGAGCCCTGGATACCAA	60.0	99 bp	Mouse
	**R:** ACTTCCAACCCAGGTCCTTC	60.3		
*Nlrp3*	**F:** AGCCCTCCTTCACCATCAG	60.2	68 bp	Mouse
	**R:** CACAAGCCTTTGCTCCAGA	60.1		
*Cas-3*	**F:** AATAGCCCTGCAGCCCAT	60.6	64 bp	Mouse
	**R:** GAGCACAGTCTCCCTGAGGAT	60.8		
*Mtor (mTORC1)*	**F:** GATAATTGCTGCTCCTGTGC	60.2	68 bp	Mouse
	**R:** AGATCTGCTCCAAGGTGGAC	60.8		
*Bim*	**F:** CACCTGCTGTGTGCTTCCTA	60.0	89 bp	Mouse
	**R:** GCTGGCCTAAAGCAGTGAAC	60.0		
*β-actin*	**F:** GATCATTGCTCCTCCTGAGC	60.0	83 bp	Mouse
	**R:** ACATCTGCTGGAAGGTGGAC	60.0		
*Nrf-2*	**F:** CGGCTCAGCACCTTGTATCT	60.4	100 bp	Mouse
	**R:** CATCTCTGGTTTGCTGCAGA	60.1		

### 2.13. Statistical Analysis

All data are presented as mean ± standard deviation (SD), and the statistical analyses were performed in GraphPad Prism (Version 9.0). For single‐endpoint outcomes measured once at necropsy, the groups were compared using one‐way ANOVA with Tukey′s multiple comparisons test, with prespecified contrasts including juglone versus CCl_4_, silymarin versus CCl_4_, vehicle versus normal, and juglone versus silymarin. Statistical significance was assessed by one‐way ANOVA, two‐way ANOVA, and Tukey′s post hoc test. A *p* value of < 0.05 was considered statistically significant.

## 3. Results

### 3.1. Body Weight and Gross Liver Findings

**Figure 1:**
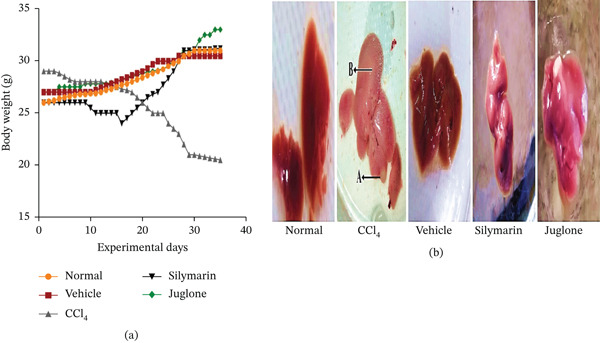
(a) Mean body weight (g) across all groups. Mice in the carbon tetrachloride (CCl₄) group showed significant weight loss compared with the normal and vehicle groups, whereas those treated with silymarin or juglone demonstrated recovery following treatment (*p* < 0.05). (b) Gross liver morphology at necropsy. Livers from the toxin group exhibited irregular, pale surfaces with visible lesions (A) and coarse scarring (B), whereas the silymarin‐ and juglone‐treated groups displayed fewer lesions and smoother surfaces, consistent with hepatoprotective effects.

Across the experimental schedule, CCl₄ exposure produced a clear systemic effect that was reflected in the body‐weight trajectory. The CCl₄ group showed a progressive decline in mean body weight from the second week, whereas the normal and vehicle groups remained comparatively stable. Both the silymarin and juglone groups experienced weight loss during the injury phase but began to recover once treatment commenced. The juglone group, in particular, showed a steady upward trend in body weight toward the later study days (Figure 1a). Some baseline body‐weight differences were observed at the start, which may have contributed to variability in the overall magnitude of change across groups. Gross liver morphology at necropsy reflected a similar injury–recovery pattern. Normal livers appeared smooth and uniformly reddish‐brown, whereas the CCl₄‐exposed livers were irregular and pale and showed coarse surface scarring. The vehicle group showed morphology closer to the normal, whereas both the silymarin‐ and juglone‐treated groups displayed fewer visible surface lesions and a more homogenous color than the CCl₄ group (Figure 1b). These findings align with a lower degree of gross hepatic injury in the treated cohorts.

### 3.2. Serum Biochemical Analysis

**Figure 2:**
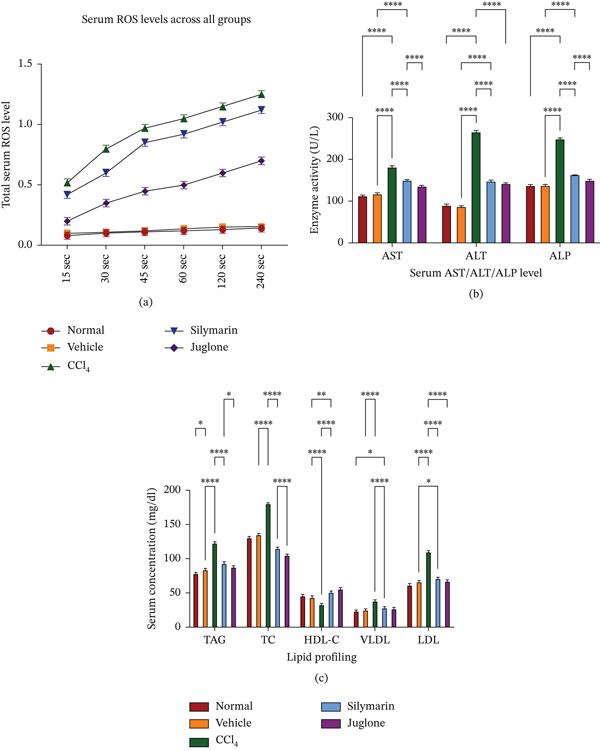
(a) Kinetic measurement of serum ROS levels at 15, 30, 45, 60, 120, and 240 s across all experimental groups, showing sustained elevation in the CCl₄ group and significant reduction with silymarin and juglone treatment. (b) Liver enzyme activities (AST, ALT, and ALP) measured in serum. CCl_4_ exposure induced significant elevation in liver enzymes, indicating hepatocellular damage. Silymarin and juglone significantly reduced enzyme levels. (c) Lipid profile analysis shows serum concentrations of TAG, TC, HDL‐C, VLDL, and LDL‐C. The CCl₄ group showed marked dyslipidemia, whereas treatment with silymarin or juglone restored lipid levels toward near‐normal values. Data are expressed as mean ± SD. Statistical comparisons were performed using two‐way ANOVA followed by Tukey′s post hoc test ( ^∗^ *p* < 0.05,  ^∗∗^ *p* < 0.01,  ^∗∗∗^ *p* < 0.001,  ^∗∗∗∗^ *p* < 0.0001).

To capture oxidative‐stress dynamics rather than a single endpoint, serum ROS was assessed kinetically at 15, 30, 45, 60, 120, and 240 s (Figure 2a). The CCl₄ group showed sustained elevation across the measurement window, whereas both silymarin and juglone groups showed lower ROS trajectories compared with CCl₄. CCl₄ exposure increased serum AST, ALT, and ALP activities relative to normal and vehicle (Figure 2b). Both silymarin and juglone shifted these enzymes activities downward compared with CCl₄ (Figure 2b), supporting an improvement in the biochemical injury signal at the terminal endpoint. In the same animals, CCl₄ produced a dyslipidemia pattern with increased TAG and TC and altered lipoprotein fractions. Silymarin and juglone moved the lipid profile toward the normal range across these parameters (Figure 2c).

### 3.3. Liver Histological Analysis

**Figure 3:**
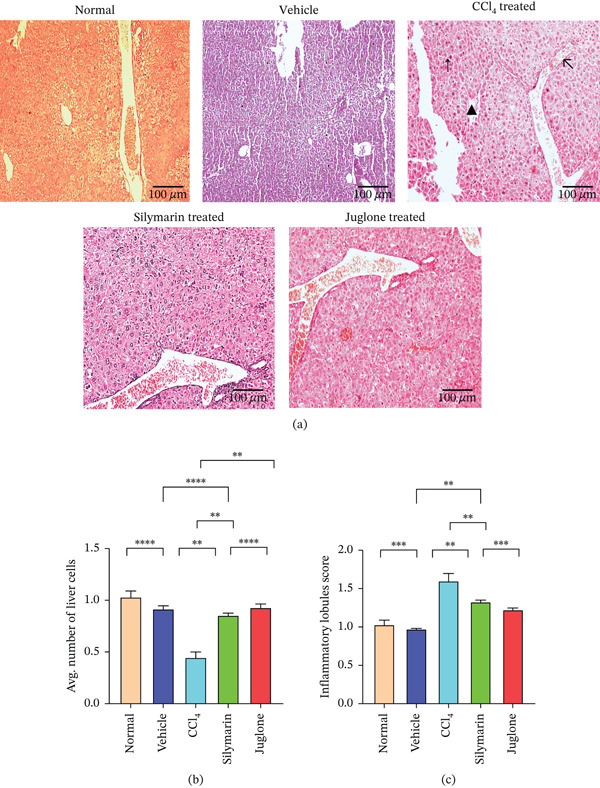
(a) Representative photomicrographs of H&E‐stained liver sections (10× magnification) from each experimental group. The normal group shows intact hepatic cords and well‐defined central veins, whereas the vehicle group displays near‐normal architecture. The CCl₄ group exhibits disrupted lobular organization with inflammatory infiltrates (↑, ↖) and ballooned hepatocytes (▲). Silymarin shows reduced inflammation and limited necrosis; Juglone shows marked histological recovery with re‐organized cords. (b) Quantitative analysis of average number of liver cells per field. CCl₄ exposure significantly reduced hepatocyte count compared to normal and treatment groups, whereas both silymarin and juglone treatment restored hepatocyte numbers. (c) Inflammatory lobule score across experimental groups. CCl₄ administration significantly increased hepatic inflammation relative to normal. Juglone and silymarin treatments both resulted in significantly reduced inflammation scores. The quantitative analysis was performed using Image J software and statistical significance determined via one‐way ANOVA with post hoc Tukey′s test. Data are presented as mean ± SD;  ^∗^ *p* < 0.05,  ^∗∗^ *p* < 0.01,  ^∗∗∗^ *p* < 0.001,  ^∗∗∗∗^ *p* < 0.0001.

Histological examination showed that the normal group retained normal hepatic cords and a well‐defined lobular pattern, with the vehicle group closely resembling this baseline structure. In contrast, the CCl₄ group exhibited disrupted lobular organization, inflammatory cell infiltration, and hepatocellular ballooning (Figure 3a). Treatment with silymarin reduced inflammatory changes and partially restored tissue continuity, whereas juglone produced comparable improvements, showing fewer infiltrates and clearer lobular boundaries relative to the toxin group. These qualitative observations were supported by quantitative analysis: The mean number of hepatocytes per field was markedly reduced in the CCl₄ group but increased in both treatment groups toward normal levels (Figure 3b). Similarly, the lobular inflammation score was elevated in the CCl₄ group and declined following silymarin or juglone treatment (Figure 3c).

### 3.4. Inflammation, Pyroptosis, and Stress Transcripts (mRNA)

**Figure 4:**
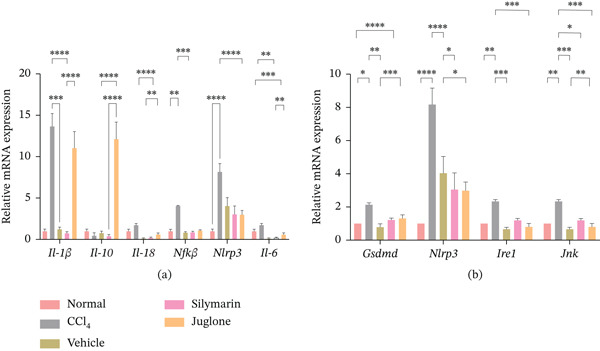
(a) Relative mRNA expression of pro‐inflammatory cytokines (*Il-1β*, *Il-6*, *Il-18*, *Nf-κb*, and *Nlrp3*) in liver tissues from CCl_4_, juglone, silymarin, and vehicle‐treated groups. Elevated levels of *Il-1β* and *Il-6* were observed in the CCl_4_ group, with significant downregulation upon treatment with juglone and silymarin. (b) Relative mRNA expression of pyroptosis‐related genes (*Gsdmd*, *Nlrp3*, *Ire1*, and *Jnk*) in liver tissues. The CCl_4_ group showed significant upregulation of *Gsdmd* and *Nlrp3* compared to normal. Juglone and silymarin treatments significantly reduced the expression of these markers, indicating inhibition of pyroptotic pathways ( ^∗^ *p* < 0.05,  ^∗∗^ *p* < 0.01,  ^∗∗∗^ *p* < 0.001,  ^∗∗∗∗^ *p* < 0.0001). Data are presented as mean ± SD. Statistical significance was assessed by two‐way ANOVA and Tukey′s post hoc test.

At the transcript level, RT‐qPCR showed that the CCl₄ model increased hepatic expression of inflammatory cytokine and signaling genes (*Il1b*, *Il6*, *Il18*, *Nfkβ*, and *Nlrp3*) relative to normal, whereas the vehicle group remained broadly comparable to baseline. Both juglone and silymarin reduced the CCl₄‐associated elevations across these inflammatory targets (Figure 4a). In parallel, the pyroptosis‐related panel showed increased expression of *Gsdmd* and *Nlrp3* along with *Ire1* and *Jnk* in the CCl₄ group, and both treatments lowered these transcripts toward baseline (Figure 4b).

**Figure 5:**
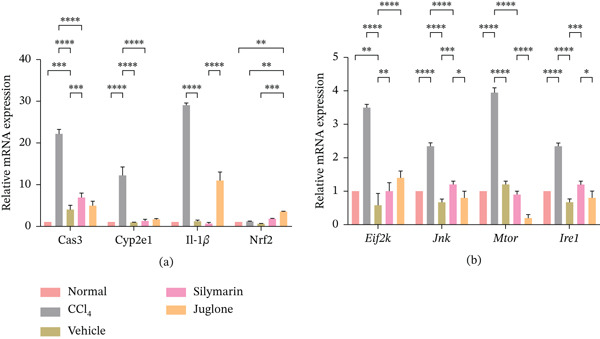
(a) Relative mRNA expression of oxidative stress‐related genes (*Casp3*, *Cyp2e1*, *Il-1β*, and *Nrf2*) in liver tissues. CCl_4_ exposure greatly expressed these genes, and these genes were effectively downregulated by the action of juglone implying that these genes possess strong antioxidant activity. (b) Relative mRNA expression of ER stress and autophagy‐associated genes (*Eif2k*, *Jnk*, *Mtor*, and *Ire1*) in liver tissues. The expression of CCl_4_ was significantly reduced in the group that had received the juglone that exhibited the potential of increased autophagic regulation. Data were presented as mean ± SD. Two‐way ANOVA and Tukey post hoc were used to assess the statistical significance:  ^∗^ *p* < 0.05,  ^∗∗^ *p* < 0.01,  ^∗∗∗^ *p* < 0.001,  ^∗∗∗∗^ *p* < 0.0001.

CCl₄ injury also produced a broad transcriptional signature consistent with cellular stress, including altered expression of *Casp3*, *Cyp2e1*, *Il1b*, and *Nrf2* relative to normal (Figure 5a). Juglone and silymarin shifted this pattern toward normal across multiple targets (Figure 5a). ER stress and autophagy‐associated transcripts (*Eif2k*, *Jnk*, *Mtor*, and *Ire1*) were elevated in the CCl₄ group and shifted downward with juglone and silymarin (Figure 5b).

### 3.5. Apoptosis and Remodeling Transcripts (mRNA)

**Figure 6:**
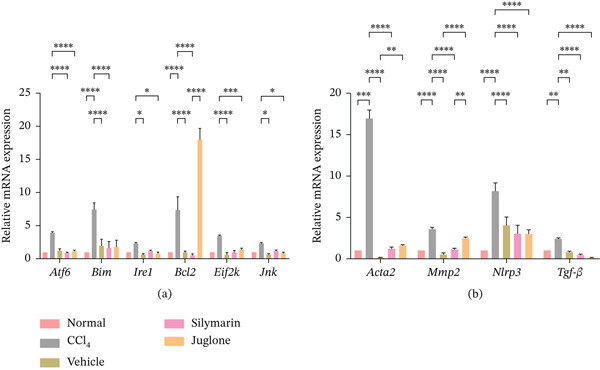
(a) Relative mRNA expression of apoptosis‐related genes. Pro‐apoptotic *Bim* was significantly upregulated in the CCl_4_ group and significantly reduced in both juglone and silymarin‐treated groups. Anti‐apoptotic *Bcl2* expression was significantly reduced in the CCl_4_ group but preserved in treated groups, suggesting protection against apoptotic cell death. (b) Relative mRNA expression of fibrotic markers (*Acta2*, *Mmp2*, *Nlrp3*, and *Tgf-β*). These genes were significantly upregulated in the CCl_4_ group, with significant downregulation observed in both juglone and silymarin‐treated groups. Data are presented as mean ± SD. Statistical significance was evaluated using two‐way ANOVA and Tukey′s post hoc test:  ^∗^ *p* < 0.05,  ^∗∗^ *p* < 0.01,  ^∗∗∗^ *p* < 0.001,  ^∗∗∗∗^ *p* < 0.0001.

The CCl₄ model increased the pro‐apoptotic marker *Bim* and suppressed the anti‐apoptotic marker *Bcl2* relative to normal. Juglone and silymarin shifted these apoptosis‐related transcripts toward baseline, with *Bcl2* preservation most prominent in the juglone group (Figure 6a). In the remodeling panel, *Acta2*, *Mmp2*, *Nlrp3*, and *Tgf-β* were increased in the CCl₄ group and reduced with juglone and silymarin (Figure 6b).

## 4. Discussion

While the hepatoprotective potential of juglone has been examined in previous in vivo fibrosis studies, several knowledge gaps remain that this work aims to address. Earlier investigations, such as those by Eidi et al. [19], primarily employed rat models treated with broad walnut leaf extracts rather than the purified compound, with outcomes focused largely on general antioxidant activity. Similarly, Zhou et al. [18] reported antifibrotic effects of juglone but emphasized the TGF‐*β*/Smad signaling axis without considering upstream cellular stress responses.

In the present study, we used a C57BL/6 mouse model and administered purified juglone derived from *R. japonica* via intraperitoneal injection to achieve consistent systemic exposure. Beyond assessing fibrosis, this study expands on previous findings by mapping a broader molecular framework that connects oxidative injury to fibrosis, ER stress, and NLRP3 inflammasome‐mediated pyroptosis, an interplay not yet comprehensively characterized in relation to juglone′s hepatoprotective activity [18, 19].

The primary objective of this study was to assess the hepatoprotective potential of the natural naphthoquinone juglone in a mouse model of CCl₄‐induced oxidative and inflammatory liver injury. In this well‐established model, cytochrome P450‐mediated metabolism of CCl_4_, predominantly via CYP2E1, generates reactive trichloromethyl radicals that trigger lipid peroxidation and tissue damage [20]. Juglone treatment was associated with improvements in both systemic and hepatic parameters. By the second week, mice in the injury group showed characteristic body weight loss, reflecting metabolic stress and systemic inflammation typical of this model [23]. Administration of juglone reversed this trajectory, producing a sustained recovery in body weight relative to the vehicle and normal (Figure 1a).

These physiological improvements were supported by histological findings. Liver sections from the CCl₄‐exposed group showed classic features of injury, including lobular disorganization, inflammatory infiltrates, and hepatocellular ballooning. Treatment with juglone was associated with a marked reduction in inflammation scores and restoration of hepatic architecture, comparable to the effects observed in the silymarin group (Figure 3a–c). At the molecular level, these structural changes corresponded with decreased expression of fibrosis‐related mRNA markers, including *Acta2* (encoding *α*‐SMA) and *Mmp2*. The downregulation of these genes suggests reduced HSC activation and a dampened profibrogenic response, consistent with antifibrotic effects previously reported in rat models [18, 24]. Biochemical analyses further corroborated the hepatoprotective role of juglone. The d‐ROMs assay, used to monitor serum ROS levels as a kinetic measure of oxidative stress, showed a significant reduction in the juglone‐treated group (Figure 2a), consistent with the restoration of membrane integrity markers (ALT, AST, and ALP).

Because ROS‐driven oxidative stress is closely linked with mitochondrial dysfunction, inflammation, and tissue injury, including in experimental organ‐injury models, the lowering of these systemic markers, coupled with the correction of CCl₄‐induced dyslipidemia (Figure 2c), suggests that juglone may improve hepatic lipid handling by mitigating the underlying oxidative burden [25–26]. Furthermore, we observed a downregulation of *Cyp2e1* mRNA in the treated groups. While we cannot confirm enzyme activity levels from transcripts alone, a reduction in *Cyp2e1* expression may be associated with a reduced potential for bioactivation of residual toxin or reduced oxidative stress generation via this P450 isoform [27, 28].

A central aspect of our investigation was the potential modulation of inflammatory cell death. The CCl_4_ injury induced a robust upregulation of pro‐inflammatory transcripts (*Il1b*, *Il6*, *Il18*, and *Nfkβ*) and pyroptosis‐related genes (*Nlrp3* and *Gsdmd*). Juglone treatment was associated with a significant downregulation of these targets (Figure 4a–b). Unlike apoptosis, pyroptosis is a lytic, highly inflammatory form of cell death driven by the NLRP3 inflammasome and Gasdermin D (GSDMD) pores [29, 30]. Although we did not measure cleaved GSDMD or active Caspase‐1 protein levels which limits our ability to confirm the execution of pyroptosis, the transcriptional suppression of *Nlrp3* and *Gsdmd* strongly implies a dampening of the priming phase of this pathway. Interestingly, regarding cytokine regulation, we noted that while silymarin normalized the anti‐inflammatory cytokine *Il10*, its levels remained elevated in the juglone group. The sustained expression of *Il-10* may reflect a distinct immunomodulatory process in which juglone supports a prolonged resolution phase, rather than simply returning the system to its baseline state.

In parallel, the molecular findings showed reduced organelle stress. Previous studies suggest that oxidative stress can interact with ER stress and autophagy through a feedback mechanism, shaping cellular responses [31]. In our research, the injury group showed increased levels of transcripts for ER stress sensors (*Atf6*, *Perk*, and *Ire1*) and the pro‐apoptotic mediator *Bim*. Juglone treatment shifted this profile, associating with downregulated ER stress markers and an upregulated Bcl2/Bim ratio (Figure 5a–b). Reduced *Perk* and *Ire1* expression is consistent with the restoration of protein‐folding homeostasis. Furthermore, the concurrent increase in *Nrf2* expression in the juglone group supports the hypothesis that this compound may bolster antioxidant defenses, thereby interrupting the cycle where ROS exacerbates ER dysfunction [32]. While we have not performed knockout studies to prove causality between NRF2 signaling and ER stress mitigation in this specific model, the gene expression pattern is consistent with established hepatoprotective networks described in the literature [31].

## 5. Conclusions

The study evaluated juglone in a CCl_4_‐based mouse liver injury model and found an overall improvement in biochemical injury readouts, lipid imbalance, systemic oxidative signal, and liver architecture, supporting a hepatoprotective association in this toxic‐injury setting. The transcriptional profile further showed downregulation of inflammatory and pyroptosis‐related transcripts together with moderation of ER stress and UPR–autophagy‐linked transcripts, which is consistent with known stress–inflammation biology in hepatic injury but should still be interpreted as gene‐level associations at this stage. Importantly, the decrease in *Acta2*, *Mmp2*, and *Tgfβ1* supports downregulation of profibrotic transcripts rather than confirmed antifibrotic activity, which would require collagen‐focused structural and protein validation. In future, the protein level validation can strengthen the study. Overall, in this work, we measured these pathway‐related changes at the mRNA level, so the results should be read in that context. The patterns we see match the shifts in serum markers and the tissue findings, but mRNA alone cannot fully confirm what is happening at the protein or activity level.

**Nomenclature**
ALP: alkaline phosphatase
ALT: alanine aminotransferase
AST: aspartate aminotransferase
Bcl2: B‐cell lymphoma 2
CCl₄: carbon tetrachloride
CYP2E1: cytochrome P450 2E1
ER: endoplasmic reticulum
GSDMD: gasdermin D
HDL‐C: high‐density lipoprotein cholesterol
H&E: hematoxylin and eosin
LDL‐C: low‐density lipoprotein cholesterol
MMP2: matrix metalloproteinase 2
NLRP3: NOD‐like receptor family pyrin domain containing 3
NQO1: NAD(P)H quinone dehydrogenase 1
NRF2: nuclear factor erythroid 2‐related factor 2
ROS: reactive oxygen species
RT‐qPCR: reverse transcription quantitative polymerase chain reaction
TAG: triglycerides
TC: total cholesterol
TGF‐*β*1: transforming growth factor beta 1
VLDL: very low‐density lipoprotein

## Funding

This study was supported by Quaid‐i‐Azam University, Islamabad, URF 2020‐2021, and the National Research Foundation of Korea, 10.13039/501100003725, RS‐2025‐25416339.

## Ethics Statement

The animal experiments were approved by the Animal Bioethics Committee of Quaid‐i‐Azam University (BEC‐FBS‐QAU2020‐271), Islamabad, Pakistan, and all procedures followed applicable guidelines and regulations.

## Conflicts of Interest

The authors declare no conflicts of interest.

## Data Availability

The data that support the findings of this study are available from the corresponding author upon reasonable request.
